# Nurturing protectors: Macrophages in the human pancreatic islet

**DOI:** 10.1016/j.stem.2024.10.006

**Published:** 2024-11-07

**Authors:** Christopher Z.W. Lee, Francesca M. Spagnoli

**Affiliations:** 1 Centre for Gene Therapy and Regenerative Medicine, King’s College London, London, Great Maze Pond, London SE1 9RT, UK

## Abstract

Two recent publications in *Cell Stem Cell*, Yang et al.^1^ and Migliorini et al.,^2^ utilized pluripotent stem cell-derived co-culture systems to explore the role of macrophages within the pancreatic islet during development and disease states.

The pancreas is a heterocrine gland, responsible for the exocrine secretion of digestive enzymes in the duodenum and the endocrine secretion of various essential blood glucose-regulating hormones. The exocrine compartment of the pancreas comprises most of the tissue (>95%), whereas endocrine cells are scattered throughout and around the remaining tissue, arranged in distinctive structures known as the islets of Langerhans. These islets constantly monitor and adjust blood glucose levels via the production of glucagon from α cells, insulin from β cells, and, to a lesser extent, somatostatin from δ cells.^3^

Immune cells, including macrophages, are also present in the pancreatic islets from the embryonic stage onward as part of the islet microenvironment.^4,5^ Besides the interplay between the immune system and pancreatic β cells in the context of type 1 diabetes (T1D),^6^ little is known about this intercellular crosstalk in other contexts. In this issue of *Cell Stem Cell*, Yang et al.^1^ and Migliorini et al.^2^ independently developed human pluripotent stem cell (PSC)-derived macrophage-containing islet culture systems, albeit using them to answer distinct questions in disease and development, respectively.

In the study by Yang et al.,^1^ the authors developed a vascularized macrophageislet (VMI) organoid model for studying macrophage-mediated host damage. First, using the GeoMx spatial transcriptomics platform on pancreatic autopsy samples from patients with COVID-19 and healthy controls, they unveiled an accumulation of proinflammatory macrophages within the islets of the COVID-19 pancreatic samples, alongside a decrease in the number of insulin+ endocrine cells without a decrease in the overall islet area. Next, they performed single-cell RNA sequencing on human cadaveric islets infected with either SARS-CoV-2 or coxsackievirus B4 (CVB4) viruses and noticed an upregulation of the pyroptotic pathway in the islet endocrine cells of both virus-infected groups. This finding suggested that pyroptosis might be related to the activity of inflammatory macrophages. To test this hypothesis, they generated a PSC-derived VMI organoid model consisting of endocrine cells, endothelial cells, and macrophages and confirmed that TNF superfamily member 12 (TNFSF12) and interleukin-1beta (IL1B) were synergistically responsible for macrophage-mediated β cell pyroptosis.

Migliorini et al.^2^ approached the relevance of islet-macrophage intercellular crosstalk from a developmental point of view. The authors generated a transcriptomic map of the developing fetal human pancreas—14- and 18-week post-conception (WPC)—using single-nuclei RNA sequencing, and characterized the composition and heterogeneity of the exocrine, endocrine, and hematopoietic compartments. Next, they decided to zoom in on the macrophages, as among the hematopoietic cells they scored the highest number of ligand-receptor (L:R) interactions with fetal pancreatic cells. To investigate the macrophage-endocrine cross-talk, they generated a PSC-derived islet organoid model in which PSC-derived macrophages were added to pancreatic endoderm-stage cultures, allowing for co-development of both cell types for the remainder of the differentiation. These resulting organoids, called eMAC-Endo, not only contained a higher number of β cells but were also capable of secreting human C-peptide, a by-product of insulin cleavage and release, upon engraftment into immunocompromised mice. This secretion was not observed in mice that were engrafted with pancreatic organoids devoid of macrophages.

Given the importance of the islets of Langerhans in glycemic control, it is unsurprising that tremendous effort has been put into understanding the development, maturation, and dysfunction of these cells. Over the past decades, mouse genetics studies have provided a wealth of information on cell-intrinsic regulators of pancreatic endocrine development and islet cell identity maintenance.^7^ However, these cells do not exist in isolation and are supported by a wide variety of other cell types, such as the mesenchyme, vasculature, infiltrating, and resident immune cells.^8^ As such, deciphering the specific communication and crosstalk between these supporting cells from the microenvironment and their host tissue represents the crucial next step toward a more complete understanding of islet biology. The wide-scale adoption of single-cell sequencing technology has provided some clarity toward this problem, allowing for the inference of L:R interactions between cell pairs within a tissue. However, these interactions remain difficult to test amid the complexity of an *in vivo* organ. Some progress has been made using PSC-derived *in vitro* systems, which provide a valuable human-based and minimalistic model to manipulate. Still, most differentiation protocols are designed to produce and maintain cells of a single lineage, necessitating further optimization when attempting to co-culture and study interactions between differing cell types.

These two studies together represent an exciting step forward in the development of more ‘realistic’ multicellular islet models, presenting complementary and concurring results in support of macrophage-induced improvements in β cell differentiation and expansion.^1,2^ These results are in agreement with previous animal studies that showed a reduction in β cell mass in mice deficient in macrophages.^9^ However, in Migliorini et al.^2^ the eMAC-Endo co-culture does not lead to an improved functionality of β-like cells *in vitro*, prior to engraftment, whereas the VMI organoids in Yang et al.^1^ secrete more insulin in dynamic glucose-stimulate insulin secretion (GSIS) assay compared to standard conditions. This discrepancy might be due to the differing co-culture strategies devised by the two groups. The eMAC-Endo model of Migliorini et al.^2^ is surrounded by a thick layer of macrophages, which account for almost 50% of the total organoid; whereas the macrophages in the Yang et al.^1^ VMI organoid model were interspaced and sparser throughout the organoid. Additionally, the VMI organoids include endothelial cells, whereas these are absent in the eMAC-Endo model *in vitro*. Conversely, the Migliorini et al.^2^ model has the advantage of being fully isogenic, with macrophages and islet cells generated from the same H1 human embryonic stem cell (hESC) line; whereas the Yang et al.^1^ model was generated from three different lines. This difference should be taken into consideration when interpreting results involving immune cells. Interestingly, Migliorini et al.^2^ also demonstrated that direct contact between the macrophages and the islet cells was not only important but also essential for the increase of β-like cells (C-peptide+/NKX6.1+) observed. What is more, the PSC-derived endocrine culture in indirect contact with macrophages, separated by a transwell, showed a reduction in β cell commitment when compared to endocrine cultures alone. This outcome suggests that macrophage-secreted factors alone might antagonize β cell development, whereas direct contact enhances it, opening up previously unrecognized avenues when considering cellular communication between different cell types. Moreover, grafts derived from eMAC-Endo organoids exhibited increased vessel density, compared to those from endocrine cells alone, when transplanted subcutaneously in immunocompromised mice. Overall, macrophages may play a direct or indirect role in promoting vascularization, a function that seems to be shared across different tissues.^10^

In summary, the incorporation of additional cell types into human PSC-islet models will greatly improve our understanding of the influence of cell-extrinsic signals from neighboring cells on pancreatic endocrine development, homeostatic adult tissue, and disease. Finally, PSC-islet co-culture systems, including those containing macrophages, might be relevant in future tissue-engineering strategies toward the treatment of diabetes.
